# Catecholaminergic Polymorphic Ventricular Tachycardia: Multiple Clinical Presentations of a Genetically Determined Disease

**DOI:** 10.3390/jcm13010047

**Published:** 2023-12-21

**Authors:** Stjepan Jurisic, Argelia Medeiros-Domingo, Florian Berger, Christian Balmer, Corinna Brunckhorst, Frank Ruschitzka, Ardan M. Saguner, Firat Duru

**Affiliations:** 1 Department of Cardiology, University Heart Center Zurich, University Hospital Zurich, 8091 Zurich, Switzerland; 2 Cardiogenetics—Swiss DNAlysis, 8600 Dubendorf, Switzerland; 3 Divison of Pediatric Cardiology, Pediatric Heart Center, Department of Surgery, University Children’s Hospital Zurich, 8032 Zurich, Switzerland; 4 Children’s Research Center, University Children’s Hospital Zurich, 8032 Zurich, Switzerland; 5 Center for Translational and Experimental Cardiology (CTEC), Department of Cardiology, University Hospital Zurich, University of Zurich, 8091 Zurich, Switzerland; 6 Center for Integrative Human Physiology, University of Zurich, 8091 Zurich, Switzerland

**Keywords:** catecholaminergic, polymorphic, ventricular tachycardia, ryanodine, sudden death

## Abstract

Background: Catecholaminergic polymorphic ventricular tachycardia (CPVT) is a rare, inherited heart rhythm disorder that is caused by variants in genes responsible for cardiac calcium homeostasis. The aim of this study was to analyze different genotype-specific clinical manifestations of this disease. Methods and Results: We analyzed five CPVT cases from our institution in the context of specific patient characteristics and genotype–phenotype correlations. In this cohort, three of the index patients were male. The median age at diagnosis was 11 (11–30) years, and median age at disease onset was 12 (12–33) years. Four index patients suffered from syncope, while one female index patient suffered from out-of-hospital cardiac arrest. Two index patients experienced concomitant atrial flutter and atrial fibrillation. Three patients received an implantable cardioverter defibrillator and one patient received an event recorder. All index patients had causative genetic variants in the *RYR2*-gene. Conclusions: This study presents various phenotypic presentations of patients with CPVT harboring different pathogenic variants in the *RYR2* gene, some of which have not previously been described in published studies. Syncope was the most prevalent symptom on admission. Adjustment of beta-blocker therapy may be necessary due to side effects. Moreover, our work further highlights the common occurrence of atrial tachyarrhythmias in these patients.

## 1. Introduction

Catecholaminergic polymorphic ventricular tachycardia (CPVT) is a genetically determined disease that is characterized by catecholamine-induced malignant ventricular tachyarrhythmias. Several phenotypic variants and underlying genetic causes were identified after its first description by Coumel et al. in the 1970s [1]. The two main genetic variants associated with CPVT involve the ryanodine receptor 2 (*RYR2*) and calsequestrin 2 (*CASQ2*). Other variants affecting triadin (*TRDN*), calmodulin (*CALM 1, CALM 2* and *CALM 3*) and trans-2,3-enoyl-CoA reductase like protein (*TECRL*) have also been described [2]. While *RYR2* variants cause the most common phenotype with autosomal dominant inheritance, *CASQ2* variants causing CPVT may be autosomal recessive or dominant [3,4].

Patients with CPVT can experience episodic syncope or cardiac arrest due to polymorphic or bidirectional VT during exercise or emotional stress. More than 30% of patients with CPVT have a history of malignant arrhythmias before diagnosis [5]. However, correct diagnosis can be challenging and patient numbers are most likely underestimated. Many patients experience multiple syncopal episodes before the correct diagnosis is established or are misdiagnosed as experiencing neurologic events. The diagnostic criteria for CPVT include documentation of stress-induced bidirectional or polymorphic VT in the absence of ECG abnormalities at rest in patients with a structurally normal heart [6]. The detection of pathogenic *RYR2* or *CASQ2* gene variants is also sufficient for diagnosis, even before clinical manifestation. The cornerstone of medical therapy is beta-blockers alongside the use of an implantable cardioverter defibrillator (ICD) in high-risk patients. In this study, we present and discuss different genetic variants of the *RYR2* gene causing CPVT, with various clinical manifestations.

## 2. Methods

The clinical courses of five CPVT patients from the Arrhythmia and Electrophysiology Division of the University Heart Center in Zurich were retrospectively analyzed. Data were gathered from existing medical records. The medical records, including demographic data, physical findings, 12-lead ECG, Holter-ECG, implantable cardioverter defibrillator (ICD) data, exercise testing, laboratory values, transthoracic echocardiography (TTE) and cardiac magnetic resonance imaging (CMR) findings were reviewed by experienced electrophysiologists and imaging specialists. The diagnosis of CPVT was defined in accordance with the 2015 European Society of Cardiology (ESC) criteria, as follows: (1) Presence of a structurally normal heart, normal ECG and exercise- or emotion-induced bidirectional or polymorphic VT. (2) Carriers of a pathogenic variant(s) in the genes *RyR2* or *CASQ2* [6]. Genetic analyses were performed using next generation sequencing (NGS) (TruSight Cardio Panel, Illumina, San Diego, CA, USA), Sanger sequencing or a single variant test. 

The study protocol was approved by the Zurich Cantonal Ethical Committee (approval number PB_2016-02109) and all patients gave informed consent for participation in this study.

## 3. Results

Five patients from five different families were diagnosed with CPVT. Of these patients, three patients were male. The median age at diagnosis was 11 (11–30) years, and median age at disease onset was 12 (12–33) years. All patients were diagnosed immediately after symptom onset, except for one patient who was initially misdiagnosed as having epilepsy. The symptoms that led to the diagnosis of CPVT were mostly syncope (four patients, 80%) and one cardiac arrest (20%). Family history revealed one other sudden cardiac death (SCD) case and three other CPVT patients. Four patients had sustained VT or VF at exercise testing, while one patient demonstrated no ventricular tachyarrhythmias under beta-blocker therapy. Beta-blockers were started in all patients and flecainide was also prescribed in two patients (20%). In one patient, the beta-blocker class had to be changed due to side effects (from propranolol to metoprolol). Three patients (60%) underwent ICD implantation (Table 1). One patient received an event recorder for rhythm monitoring.

**Patient 1:**  *A de novo heterozygous RYR2 variant (c.11954 T>A, NM_001035.2, p.Met3985Lys).*

The proband, a 24-year-old man, was referred initially at the age of 11 after experiencing a syncope while playing a video game. He had already experienced four episodes of syncope in previous years. Neurologic examination including electroencephalography and cerebral magnetic resonance imaging yielded no abnormalities. During exercise testing, the patient developed bidirectional VT that degenerated into ventricular fibrillation, which was then defibrillated successfully. No abnormalities were detected on ECG, TTE or CMR. The patient underwent implantation of a dual-chamber ICD with epicardial electrodes. The initial medication was propranolol 30 mg tid, which was later changed to metoprolol 100 mg qd and flecainide 50 mg bid. Propranolol use resulted in nightmares and sleeping disorders and, therefore, it was changed to metoprolol, which was better tolerated by the patient. 

**Figure 1:**
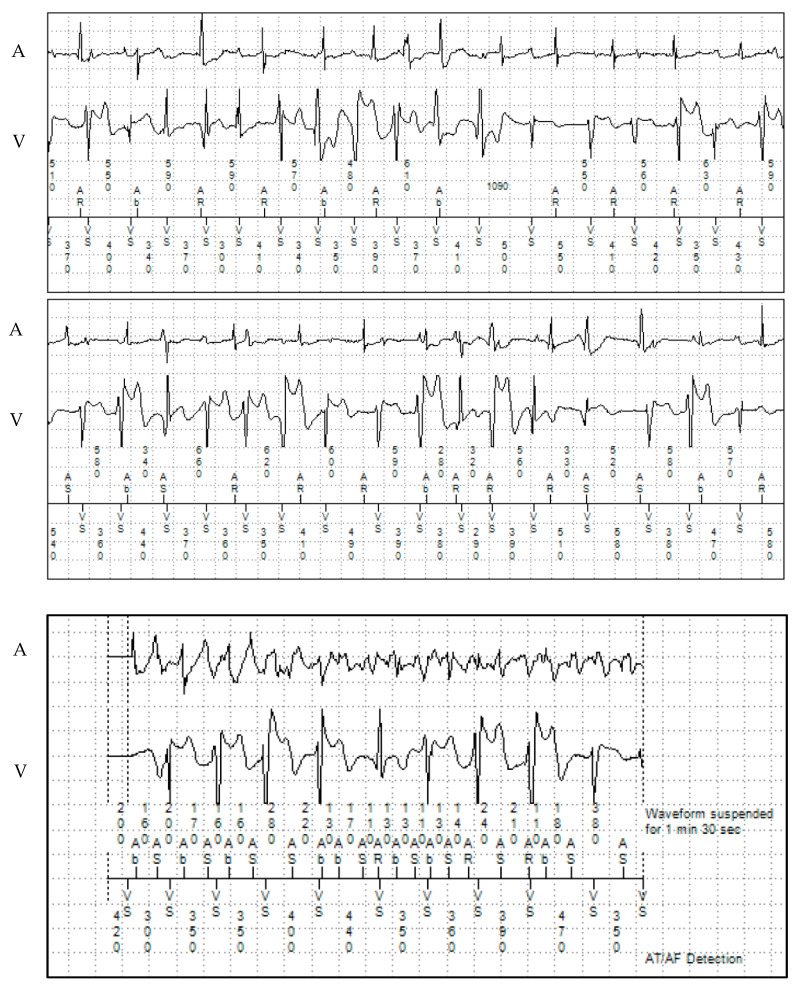
Ventricular and later sustained arrhythmia episodes in patient 1 documented on his dual-chamber ICD with remote monitoring. A: atrial electrogram; V: ventricular electrogram.

**Figure 2:**
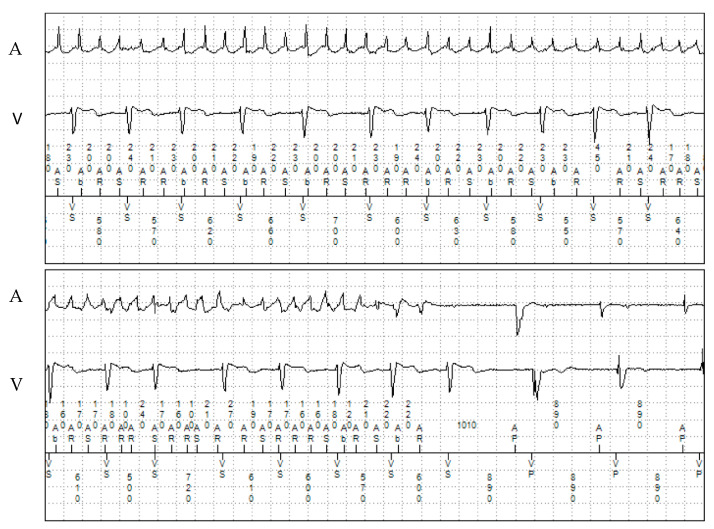
Monitored episode of atrial fibrillation in patient 1. A: atrial electrogram; V: ventricular electrogram.

Genetic screening (Sanger sequencing) showed a *RYR2* variant of uncertain significance (VUS) (c.11954 T>A; p.Met3985Lys). Genetic screening of both of the patient’s parents and his sister were negative, suggesting a de novo variant or a different father in the index patient. The patient had additional paroxysmal atrial fibrillation (AF) and atrial flutter (partially with 2:1 conduction), which started at the age of 18. The longest detected episode of an atrial tachyarrhythmia during remote monitoring was 1 h 21 min. (Figure 1 and Figure 2). The patient remained asymptomatic for these atrial arrhythmias. No oral anticoagulation was prescribed as he had a CHA_2_DS_2_-VASc score of 0.

**Patient 2:**  *A likely pathogenic heterozygous RYR2 variant (c.14246G>A, NM_001035.3, p.Gly4749Glu).*

**Figure 3:**
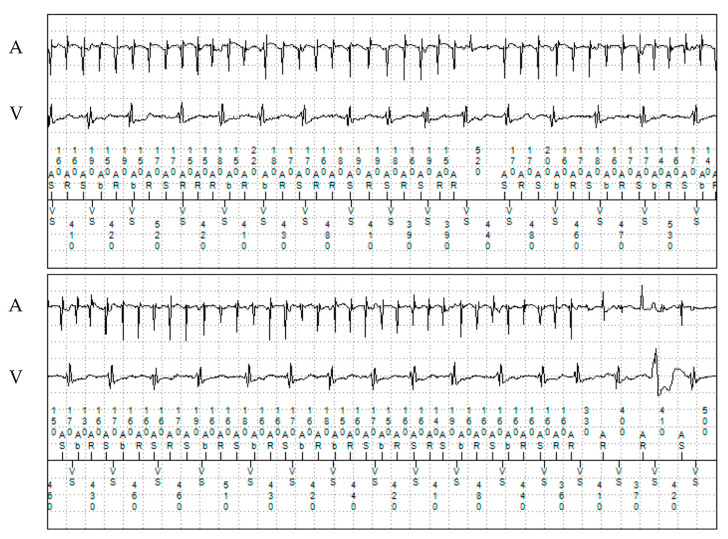
Episode of atrial fibrillation on remote monitoring with the dual-chamber ICD in patient 2. A: atrial electrogram; V: ventricular electrogram.

**Figure 4:**
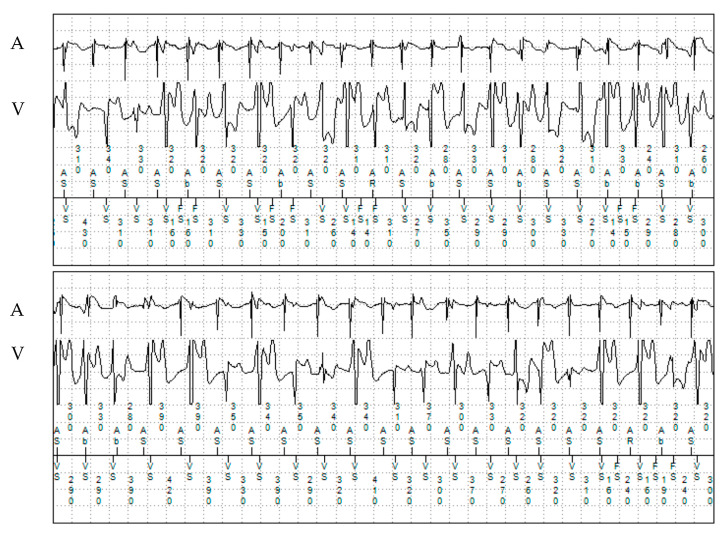
Episode of atrial tachycardia and ventricular ectopy in patient 2. A: atrial electrogram; V: ventricular electrogram.

The proband, a 31-year-old man, suffered a syncope during physical activity and was diagnosed with CPVT at the age of 12. TTE showed a structurally normal heart. He underwent implantation of a dual-chamber epicardial ICD. The patient underwent refixation of the pleural defibrillation electrode one year after implantation due to system dysfunction. At the age of 18, the patient experienced ventricular fibrillation (VF), which was adequately detected and successfully terminated by the implanted device. After this episode, propranolol dosage was increased, with no recurrent ventricular tachyarrhythmias occurring since then. Multiple asymptomatic episodes of paroxysmal AF were detected with remote monitoring (Figure 3 and Figure 4). No oral anticoagulation was prescribed, given his CHA_2_DS_2_-VASc score of 0.

Genetic testing (Trusight Cardio Panel) confirmed the presence of a heterozygous likely pathogenic variant in *RYR2* (c.14246G>A p.(Gly4749Glu). Genetic testing of the patient’s parents and sister was not possible. The patient was prescribed propranolol 50 mg tid and has since remained asymptomatic.

**Patient 3:**  *A pathogenic heterozygous RYR2 variant (c.1069G>A, NM_001035.3, p.Gly357Ser).*

**Figure 5:**
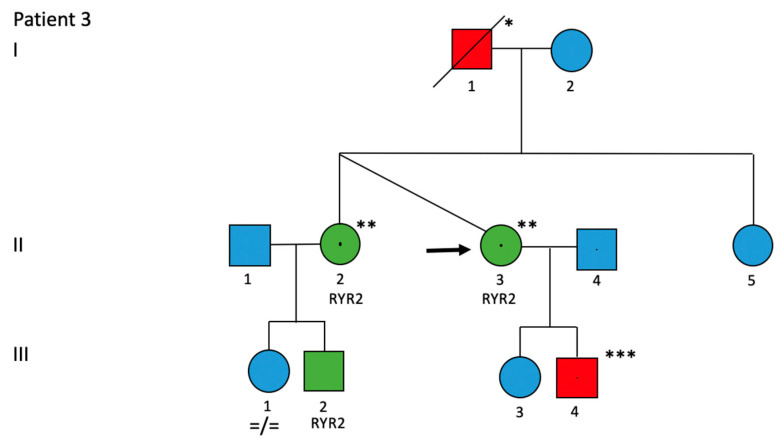
Patient 3, *RYR2*: c.1069G>A p.(Gly357Ser). Roman numerals indicate each generation; Arabic numbers indicate each individual; black arrow indicates index patient; circle and box indicate female rather than male gender; green box indicates phenotypically affected family member; red boxes and circles are family members with suspected phenotypes; blue boxes and circles are family members without clinical phenotypes; slashes indicate deceased family members; each genetic variant is listed above; =/= indicates non-presence of variant/variant. * died at age 57 unexpectedly; ** bizygotic; *** not genetically tested yet, but often has palpitations.

The proband, a 45-year-old woman, was referred to our department following physical stress-related syncope. CMR revealed normal left ventricular systolic function and no structural abnormalities. Coronary artery disease was ruled out on coronary CT angiography. She was diagnosed with CPVT following the detection of a pathogenic (Class V) heterozygous variant in *RYR2* (c.1069>A p.Gly357Ser) (single variant test). The patient had a positive family history, with a dizygotic twin sister with the same genetic variant who had remained asymptomatic while taking a beta-blocker (Figure 5). The patient’s nephew (the son of her twin sister) survived sudden cardiac arrest and underwent implantation of a subcutaneous ICD before diagnosis of our proband. Her nephew later had the same variant detected as our proband. The patient received an implantable cardiac loop monitor with home monitoring, which revealed no further arrhythmic episodes while on bisoprolol. Stress ergometry under bisoprolol revealed no ventricular tachyarrhythmias.

**Patient 4:**  *A novel RYR2 variant with uncertain significance (c.5648C>A, NM_001035.2, p.Ala1883Asp).*

The proband, a 22-year-old man, suffered from recurrent syncope during exercise at the age of seven and experienced an almost fatal drowning accident. The patient described the episodes as following: “First I don’t see well, I see black dots, then I am gone and wake up again. It is like turning a light switch on.” Because the patient’s mother suffered from epilepsy, extensive neurologic examinations were carried out. These examinations, including EEG, were unremarkable. CPVT was diagnosed 2 years later, following positive exercise testing with sustained polymorphic VT. TTE showed no abnormalities. Genetic testing (Sanger sequencing) revealed a novel heterozygous *RYR2* variant (c.5648C>A, NM_001035.2, p.Ala1883Asp). This variant was also present in the patient’s mother, masking her CPVT as epilepsy, and was not detected in the father. Otherwise, the family history was unremarkable and no further genetic testing was carried out in other relatives. Due to persistent ventricular arrhythmias during exercise under propranolol therapy, the medical therapy was changed to metoprolol along with flecainide, with subsequent regression of ventricular arrhythmias during stress testing. ICD implantation was extensively discussed with the patient who was very reserved about the device. In regular Holter recordings, no high-grade ventricular arrhythmias were detected since then.

**Patient 5:**  *A likely pathogenic RYR2 variant (c.6682G>T, NM_001035.3, p.Gly2228Cys).*

A 30-year-old female patient was diagnosed with CPVT after an out of-hospital cardiac arrest due to polymorphic VT. The incident occurred during a dispute with her supervisor. Coronary CT angiography showed no abnormalities. A CMR performed 6 days after resuscitation revealed mildly reduced left ventricular systolic function but no signs of fibrosis or other structural heart disease. A TTE showed mildly reduced left ventricular systolic function with a light midseptal hypokinesia and CMR also confirmed these findings. The right ventricle was normal in volume and systolic function. Given the good neurologic outcome, the patient received a subcutaneous ICD and started on bisoprolol 10 mg daily and lisinopril 5 mg daily. Genetic testing (Trusight Cardio Panel) revealed a likely pathogenic heterozygous variant in exon 43 of the *RYR2* genec.6682G>T (p. Gly2228Cys), which has not been previously described. However, on this highly conserved amino acid position of the *RYR2* gene, other likely pathogenic variants have already been described. In addition, this variant was detected using three different prediction programs (MutationTaster, SIFT and PolyPhen). However, based on the current knowledge, a genetic cause for dilative cardiomyopathy could neither be confirmed nor definitely excluded. Follow-up echocardiography 2 years later revealed left ventricular function at the lower limit of the norm with no signs of structural heart disease. Therefore, the CPVT diagnostic criterion of a structurally normal heart was fulfilled. We interpret the CMR scan findings in the course of post-resuscitation left ventricular dysfunction. Reversible reduction of left ventricular dysfunction after successful resuscitation from cardiac arrest is a known phenomenon. The patient did not receive any ICD therapies during follow-up. The father of the patient had a dilated cardiomyopathy with an implanted ICD after experiencing a VF episode and genetic screening was denied. Her two brothers were asymptomatic and did not undergo genetic testing.

### Long-Term Follow-Up of the Patient Cohort

The median follow-up of our patients with regular check-ups in our Channelopathy Outpatient Clinic was 5 years (IQR 2.3–6.7 years). During this time, none of our patients experienced sustained ventricular arrhythmias or required ICD therapy. No patient died during follow-up. Patients 1 and 2 presented with paroxysmal sustained atrial arrhythmias that were documented with remote monitoring but remained asymptomatic. Therefore, no specific antiarrhythmic treatment for these atrial arrhythmias was carried out. 

## 4. Discussion

A hallmark of CPVT is bidirectional or polymorphic VT induced by increased sympathetic tone due to physical or emotional stress. Despite identification of genetic variants responsible for disease and recent progress in its understanding, there are still multiple gaps in our knowledge. In this study, we describe five patients with CPVT harboring rare heterozygous genetic variants in the *RYR2* gene and presenting with various clinical manifestations. Three of these variants have not been previously described in the literature. Moreover, two of the five index patients in our cohort suffered from atrial tachyarrhythmias before the age of 35.

The core of the pathogenesis in CPVT is impaired intracellular calcium handling, which is enhanced in the setting of increased sympathetic drive [7]. The occurrence of arrhythmias in CPVT is known to be due to inadequate sarcoplasmic reticulum (SR) Ca^2+^ release during diastole that opens the Na^+/^Ca^2+^ exchanger on the surface membrane. As a result, a transient inward current is created that forces Ca^2+^ out of the SR, and as a consequence, depolarizes the sarcolemma [2]. This results in increased intracellular Ca^2+^-levels, leading to delayed afterdepolarizations (DADs), and the triggering of ventricular arrhythmias [8].

A key role in CPVT pathogenesis involves the malfunction of *RYR2*, a tetrameric protein expressed on the SR membrane. Several mechanisms have been proposed and remain controversial; the first mechanism is based on an impaired Calstabin 2-RYR2 interaction, which leads to inappropriate channel opening and Ca^2+^ leak during diastole [9]. The second hypothesis is described as a store overload-induced Ca^2+^ release [10,11]. This mechanism is characterized by enhanced *RYR2* sensitivity to the level of free Ca^2+^ in the SR; subsequently, a lower threshold is required for *RYR2* activation. This may lead to a Ca^2+^ spillover from the SR. The third mechanism refers to the intradomain interactions of *RYR2* that are important for its stabilization [12]. Variants may interrupt the intramolecular structure, resulting in a diastolic leaking of Ca^2+^.

There are two genetic variants that are typical in CPVT. The most common type is caused by variants in the *RYR2* gene and accounts for 50–70% of CPVT cases. The inheritance is autosomal dominant. The recessive form of typical CPVT is caused by variants in the *CASQ2* gene, which encodes for cardiac calsequestrin [3,13]. Calsequestrin is a SR Ca^2+^ binding protein that has a function as a Ca^2+^ buffer as well as a regulatory role in inhibiting *RYR2* activity [14,15]. Recently, a new nomenclature of atypical CPVT was introduced, including variants in calmodulin (CALM1, CALM2 and CALM3), cardiac triadin (TRDN) and trans-2,3-enoyl-CoA reductase-like protein (TECRL) [2]. All of the above-mentioned variants affect the intracellular Ca^2+^ balance [2]. In general, there is a wide phenotypic overlap between typical and atypical forms [2]. Bidirectional VT is mostly observed in typical CPVT. 

It is known that CPVT patients have an increased incidence of atrial tachyarrhythmias [16,17]. A study from the Australian Genetic Heart Disease Registry described a prevalence of 21% of concomitant atrial tachyarrhythmias [18]. Data from a large international multicenter registry reported similar prevalence of atrial arrhythmias in CPVT patients [19]. Moreover, the mean age of atrial tachyarrhythmia onset in the Australian CPVT cohort was very early (age 26) [18]. However, the reported numbers are in general coherent with our observations. AF usually occurs due to triggered focal activity and reentry circuits [20]. Hove-Madsen et al. described an increase in spontaneous Ca^2+^ release from the SR in patients with atrial tachyarrhythmias [21]. King et. al. showed in electrocardiographic experiments of stimulated mice hearts, which are homozygous for the CPVT-associated variant RYR2-P2328S (RYR2^S/S^), that the atria have reduced epicardial conduction velocities and lower maximum rates of action potential depolarization compared to wild-type mice [22]. There is evidence that *RYR2* malfunction and impaired SR Ca^2+^ release play a key role in the development of atrial arrhythmias [23]. Furthermore, studies showed that mice with the absence of Calstabin 2 (also known as FKBP12.6), which is a regulatory protein important for stabilization of *RYR2*, were vulnerable to pacing-induced AF and demonstrated increased SR Ca^2+^ leak [24]. These observations support the hypothesis of atrial arrhythmia susceptibility in patients with *RYR2* variants, which we were able to confirm in our clinical study. Two of our five patients suffered from paroxysmal sustained atrial tachyarrhythmias before the age of 35, despite a structurally normal heart with normal atrial dimensions and in the absence of other typical risk factors for these arrhythmias.

Interestingly, a high heart rate in AF can trigger Ca^2+^ overload and intensified Ca^2+^ release from the SR may occur [25,26]. This may support our hypothesis, that atrial tachyarrhythmias in CPVT patients can potentially trigger ventricular arrhythmias. Although none of our patients showed sustained VT after onset of atrial tachyarrhythmia episodes, patients 1 and 2 had an increased occurrence of polymorphic ventricular extrasystoles during simultaneous atrial tachyarrhythmia episodes.

With regard to management options, beta-blockers without intrinsic sympathomimetic characteristics are considered as the “first line” medical therapy [27]. Non-selective beta-blockers (e.g., propranolol) show better characteristics compared to beta-1 selective beta-blockers in preventing ventricular arrhythmias [28]. Secondly, flecainide has been shown to exert beneficial antiarrhythmic effects. It acts as a sodium channel blocker and may inhibit *RyR2* channels, thus preventing calcium overload [29]. Moreover, sodium channel blockage may further decrease the rate of triggering beats, supporting the use of a combination of both drugs in refractory ventricular arrhythmias [30]. Interestingly, CPVT patients may show inappropriate response to beta-blockers but good therapeutic response to flecainide [31]. For CPVT patients who remain symptomatic despite maximal drug therapy, left cardiac sympathetic denervation presents a valuable option [32]. Frequent post-procedural side effects, such as unilateral hand dryness and hyperhidrosis, must be taken into consideration [32]. High-risk patients who experience cardiac arrest, syncope or sustained ventricular arrhythmias, particularly despite optimal medical therapy, need to undergo ICD implantation. ICD should be used in addition to medical therapy and not as a replacement [33].

Data on the management of genotype-positive, phenotype-negative relatives are scarce. Expert opinion supports the use of beta-blockers in the younger population. The role of beta-blocker therapy in older asymptomatic patients who are diagnosed in adulthood via genetic testing needs to be identified [34].

## 5. Conclusions

In conclusion, this study presents various phenotypic presentations of patients with CPVT harboring different pathogenic variants in the *RYR2* gene, some of which we describe for the first time in published literature. Syncope was the most prevalent symptom on admission. Moreover, our work further highlights the high prevalence of atrial tachyarrhythmias in these patients.

## Figures and Tables

**Table 1:** Clinical characteristics.

Patient No.	Age (years)	Sex	Presentation	Genetics	AF/AT	LAVI (mL/m^2^)	ICD	Type of ICD	Medication
1	24	M	syncope	RYR2 (c.11954 T>A p.Met3985Lys)	yes	32	yes	dual-chamber epicardial	metoprolol, flecainide
2	31	M	syncope	RYR2 (c.14246G>A p.(Gly4749Glu)	yes	34	yes	dual-chamber epicardial	propranolol
3	48	F	syncope	RYR2 (c.1069G>A p.(Gly357Ser)	no	22	no		bisoprolol
4	22	M	syncope	RYR2 (c.5648C>A, p.Ala1883Asp)	no	21	no		metoprolol, flecainide
5	36	F	OHCA	RYR2: c6682G>T (p.Gly2228Cys)	no	-	yes	S-ICD	none

AF/AT, atrial fibrillation/atrial flutter, atrial tachycardia; ICD, cardioverter defibrillator; LAVI, left atrial volume index; OHCA, out of-hospital cardiac arrest; RYR 2, ryanodine receptor 2; S-ICD, subcutaneous implantable cardioverter defibrillator.

## Data Availability

Upon urgent request and associated need, our data are available, while our upmost intention is to protect our patients’ privacy.
